# Association of *Clostridium butyricum* Therapy Using the Live Bacterial Product CBM588 with the Survival of Patients with Lung Cancer Receiving Chemoimmunotherapy Combinations

**DOI:** 10.3390/cancers16010047

**Published:** 2023-12-21

**Authors:** Yusuke Tomita, Shinya Sakata, Kosuke Imamura, Shinji Iyama, Takayuki Jodai, Koichi Saruwatari, Shohei Hamada, Kimitaka Akaike, Moriyasu Anai, Kazuaki Fukusima, Akira Takaki, Hirotake Tsukamoto, Yoshihiko Goto, Chihiro Motozono, Kenji Sugata, Yorifumi Satou, Takamasa Ueno, Tokunori Ikeda, Takuro Sakagami

**Affiliations:** 1 Department of Respiratory Medicine, Kumamoto University Hospital, Honjo 1-1-1, Chuo-ku, Kumamoto 860-8556, Japan; 2 Division of Clinical Immunology and Cancer Immunotherapy, Center for Cancer Immunotherapy and Immunobiology, Graduate School of Medicine, Kyoto University, Kyoto 606-8501, Japan; 3 Division of Infection and Immunity, Joint Research Center for Human Retrovirus Infection, Kumamoto University, Honjo 2-1-1, Chuo-ku, Kumamoto 860-0811, Japan; 4 Division of Genomics and Transcriptomics, Joint Research Center for Human Retrovirus Infection, Kumamoto University, Honjo 1-1-1, Chuo-ku, Kumamoto 860-8556, Japan; 5 Laboratory of Clinical Pharmacology and Therapeutics, Faculty of Pharmaceutical Sciences, Sojo University, 4-22-1 Ikeda, Nishi-ku, Kumamoto 860-0082, Japan

**Keywords:** antibiotics, CBM588, dysbiosis, gut microbiome, immune checkpoint inhibitor, *Clostridium butyricum*, lung cancer, proton pump inhibitors

## Abstract

**Simple Summary:**

Gut microbiota is a key regulators of the efficacy of immune checkpoint inhibitor (ICIs). Thus, manipulating microbiota may enhance cancer treatment outcomes. *Clostridium butyricum* MIYAIRI 588 strain (CBM588) enhances the effects of ICI monotherapy in patients with advanced lung cancer. However, its effect on the outcomes of chemoimmunotherapy combinations in lung cancer remains unknown. We retrospectively analyzed 106 patients with stage IV or recurrent metastatic non-small cell lung cancer (NSCLC) consecutively treated with chemoimmunotherapy combination. CBM588 significantly extended the overall survival in patients with NSCLC receiving chemoimmunotherapy combinations and was associated with overall survival in patients using proton pump inhibitors and/or antibiotics. CBM588-induced manipulation of commensal microbiota has the potential to enhance the efficacy of chemoimmunotherapy combinations. The survival benefit of CBM588 in the tumor-programmed cell death ligand 1 (PD-L1) < 1% cohort was higher than that in the PD-L1 1–49% and PD-L1 ≥ 50% cohorts. Further exploration of the synergy between CBM588 and immunotherapy is warranted.

**Abstract:**

The gut microbiota has emerged as a key regulator of immune checkpoint inhibitor (ICI) efficacy. Therapeutic approaches aimed at manipulating the microbiota through targeted reconstitution to enhance cancer treatment outcomes have garnered considerable attention. A single live microbial biotherapeutic bacterium, *Clostridium butyricum* MIYAIRI 588 strain (CBM588), has been shown to enhance the effects of ICI monotherapy in patients with advanced lung cancer. However, whether CBM588 affects the outcomes of chemoimmunotherapy combinations in lung cancer remains unknown. We hypothesized that CBM588 augments the effect of chemoimmunotherapy combinations and restores diminished effectiveness in patients with non-small cell lung cancer (NSCLC) receiving dysbiosis-inducing drugs. To validate this hypothesis, we retrospectively analyzed 106 patients with stage IV or recurrent metastatic NSCLC consecutively treated with chemoimmunotherapy combinations. A survival analysis was performed employing univariate and multivariate Cox proportional hazard models with inverse probability of treatment weighting (IPTW) using propensity scores. Forty-five percent of patients received *Clostridium butyricum* therapy. CBM588 significantly extended overall survival in patients with NSCLC receiving chemoimmunotherapy. The favorable impact of CBM588 on the efficacy of chemoimmunotherapy combinations varied based on tumor-programmed cell death ligand 1 (PD-L1) expression. The survival benefit of CBM588 in the PD-L1 <1% cohort was higher than that in the PD-L1 1–49% and PD-L1 ≥ 50% cohorts. Furthermore, CBM588 was associated with improved overall survival in patients receiving proton pump inhibitors and/or antibiotics. CBM588-induced manipulation of the commensal microbiota holds the potential to enhance the efficacy of chemoimmunotherapy combinations, warranting further exploration of the synergy between CBM588 and immunotherapy.

## 1. Introduction

Immune checkpoint inhibitors (ICIs) have revolutionized the treatment of advanced lung cancer [1]. Current therapeutic options for advanced non-small cell lung cancer (NSCLC) include ICI monotherapy and the combination of ICIs with platinum-containing chemotherapy, known as chemoimmunotherapy combinations (https://www.nccn.org/guidelines, accessed on 7 December 2023) [2,3]. As the synergistic efficacy of chemoimmunotherapy combinations has been shown to be beneficial in advanced cases, these combinations have become as prevalent as ICI monotherapy. Clinical guidelines now recommend chemoimmunotherapy combinations as the standard first-line treatment for NSCLC, irrespective of the expression of tumor-programmed cell death ligand 1 (PD-L1), which is a predictive biomarker in NSCLC [2,3]. Despite the availability of these treatments, their effectiveness has been limited to a subset of patients, highlighting the necessity for improved strategies aimed at reinvigorating the antitumor immune response facilitated by ICIs [4,5,6].

The gut microbiota has emerged as a key regulator of the therapeutic efficacy of ICIs [5,6,7,8,9,10]. Medication significantly influences the structure of the gut microbiome, and its overuse is associated with increased morbidity and mortality [11,12,13]. Proton pump inhibitors (PPIs) and antibiotics are frequently administered concomitantly with cancer treatment and compromise the effectiveness of ICIs, potentially by inducing gut dysbiosis [14,15,16,17,18]. These findings have prompted interest in targeting the gut microbiota to enhance ICI efficacy. Notably, accumulating clinical evidence suggests that manipulating the gut microbiota through fecal microbiota transplantation (FMT) using stools from ICI-responsive patients enables patients with melanoma to overcome primary resistance to ICIs [19,20]. These findings lend support to therapeutic strategies that target the gut microbiota through controlled reconstitution to enhance clinical outcomes in cancer immunotherapy [5,9,21].

Dizman et al. reported that a single microbial, live biotherapeutic bacterium, *Clostridium butyricum* MIYAIRI 588 strain (CBM588, MIYA-BM^®^), modulated the gut microbiome and enhance the clinical benefits of ICIs in a randomized phase IB clinical trial comparing nivolumab/ipilimumab with or without CBM588 in patients with advanced renal cell carcinoma (NCT03829111), suggesting the potential benefits of combining CBM588 with ICIs [22]. Moreover, we previously reported that patients receiving CBM588 exhibited a reduced relative abundance of potentially harmful oral bacteria in the gut and that CBM588 could potentially enhance the efficacy of ICI monotherapy in patients with advanced NSCLC [18,23]. We revealed that the administration of CBM588, especially in patients with advanced lung cancer undergoing PPI and/or antibiotic treatments, substantially restored the efficacy of ICIs and improved patient survival in a cohort primarily (>90%) receiving ICI monotherapy [18,23]. However, the impact of CBM588 on the outcomes of chemoimmunotherapy combinations, especially in the context of patients with advanced lung cancer, remains unexplored. In addition, post hoc analyses of randomized trials demonstrated the negative impact of antibiotic exposure in patients treated with ICI monotherapy compared with those in the control arm receiving conventional chemotherapy [17,24]. However, antibiotic exposure did not adversely affect clinical outcomes in patients receiving chemoimmunotherapy combinations for NSCLC [25], confirming that the detrimental effect of antibiotic exposure is limited to ICI monotherapy. Conversely, post hoc analyses of randomized clinical trials indicated that PPIs negatively influenced the magnitude of ICI efficacy in both ICI monotherapy and chemoimmunotherapy combinations [14,26,27]. These findings raise questions about whether the impact of the live bacterial product CBM588 on the efficacy of chemoimmunotherapy combinations differs from its impact on ICI monotherapy.

While CBM588 reportedly modulates the gut microbiome and enhances the clinical benefits of ICI monotherapy and dual ICIs in patients with advanced cancers, its potential influence on the outcomes of chemoimmunotherapy combinations in lung cancer remains unexplored. We hypothesized that CBM588 amplifies the efficacy of chemoimmunotherapy combinations and restores their diminished effectiveness in patients with NSCLC receiving dysbiosis-inducing drugs through the modulation of the gut microbiota. Accordingly, we conducted a retrospective study on patients with NSCLC to assess the impact of CBM588 on chemoimmunotherapy outcomes as well as its potential to restore treatment effectiveness. This study could provide valuable insights into enhancing the efficacy of chemoimmunotherapy and improving patient outcomes.

## 2. Materials and Methods

### 2.1. Patients

Patients were eligible for inclusion in the study if they had stage IV or recurrent metastatic NSCLC classified according to Tumor–Node–Metastasis (TNM) staging classification of lung cancer, ver. 8. We conducted a retrospective analysis of 106 patients with stage IV or recurrent metastatic NSCLC who were consecutively treated with chemoimmunotherapy combinations in a routine clinical setting at Kumamoto University Hospital from 1 January 2019, to 28 July 2022. Forty-nine patients received pembrolizumab (200 mg intravenously every 3 weeks) in combination with carboplatin (area under the curve 5 mg/mL per min every 3 weeks) plus pemetrexed (500 mg/m^2^ intravenously every 3 weeks). Twenty-six patients received pembrolizumab (200 mg intravenously every 3 weeks) in combination with carboplatin (area under the curve 6 mg/mL per min every 3 weeks) plus nab-paclitaxel (100 mg/m^2^ intravenously every week). Eleven patients received atezolizumab (1200 mg intravenously every 3 weeks) in combination with bevacizumab (15 mg/kg intravenously every 3 weeks) plus paclitaxel (175–200 mg/m^2^ intravenously every 3 weeks) plus carboplatin (area under the curve 6 mg/mL per min every 3 weeks). Four patients received atezolizumab (1200 mg intravenously every 3 weeks) in combination with carboplatin (area under the curve 6 mg/mL per min every 3 weeks) plus nab-paclitaxel (100 mg/m^2^ intravenously every week). Ten patients received nivolumab (360 mg intravenously every 3 weeks) plus ipilimumab (1 mg/kg intravenously every 6 weeks) in combination with platinum doublet chemotherapy. Six patients were excluded owing to insufficient clinical information at the onset of ICI treatment or an uncertain lung cancer diagnosis. The study cohort comprised 72 men and 28 women, with a median age of 67 years (interquartile range: 38–71). All patients received ICI therapy (anti-PD-(L)1 antibody) in combination with chemotherapy. Medical records were reviewed, and treatment continued until disease progression, undesirable toxicity, or consent withdrawal. We extracted the following data from the database: date, treatment details, age, sex, histology, PD-L1 status, initial diagnosis stage, Eastern Cooperative Oncology Group (ECOG) performance status (PS), smoking status, driver mutations, liver metastases, oral or intravenous PPI use within 30 days before and after the initiation of chemoimmunotherapy (for >3 weeks during immunotherapy), oral or intravenous antibiotic use within 60 days before starting chemoimmunotherapy, and *C. butyricum* therapy using CBM588 (MIYA-BM^®^, Miyarisan Pharmaceutical Co., Ltd., Tokyo, Japan) prescribed within 3 weeks before initiating chemoimmunotherapy and/or concurrently with chemoimmunotherapy until cessation [18,23]. Time frames for PPI use, antibiotic use, and *C. butyricum* therapy were determined based on prior analyses [14,15,17,18,23]. In the non-CBM588 group, three patients received probiotic therapy using *Bifidobacterium* (BIOFERMIN TABLETS^®^) during the administration of chemoimmunotherapy combinations. In the CBM588 group, the patients did not receive live bacterial product other than CBM588 during the administration of chemoimmunotherapy combinations. Patient characteristics are summarized in Table 1.

Histories of *C. butyricum* therapy, PPI use, and antibiotic use were extracted from a prescription database and manually cross-referenced with medical records. The PD-L1 tumor proportion score (TPS) was determined through PD-L1 immunohistochemistry (clone 22C3; Dako North America, Inc., Carpinteria, CA, USA).

### 2.2. Statistical Analysis

Patient characteristics were delineated based on CBM588 administration status and compared using Fisher’s exact test and the Wilcoxon rank-sum test for categorical and continuous data, respectively. Progression-free survival (PFS) and overall survival (OS) were assessed using the Kaplan–Meier method, with differences determined via two-tailed log-rank tests. PFS was measured from the date the chemoimmunotherapy combination was started to the date of documented progression or death from any cause. OS was calculated from the date of initiation of the chemoimmunotherapy combination to death from any cause or last follow-up, with a data cutoff date of April 7, 2023. The median (IQR) follow-up durations were 14.5 (8.0–27.3) and 5.5 (3.0–14.0) months for PFS and OS, respectively. Univariate Cox proportional hazard analyses using sex, age, ECOG PS, histology, smoking history, tumor PD-L1 status, initial stage, therapy line, driver mutation status, liver metastasis, antibiotic use, PPI use, and *C. butyricum* therapy using CBM588 were employed to estimate the hazard ratio (HR) and 95% confidence interval (CI). Covariate selection was based on the results of univariate Cox proportional hazard analyses, and a multivariable Cox proportional hazard analysis was conducted. Apart from these methods, survival analysis was also conducted using univariate and multivariate Cox proportional hazards regression models incorporating propensity scores to mitigate potential confounding factors affecting treatment assignment. Propensity score adjustment included sex, age, smoking history, ECOG PS, histology, tumor PD-L1 status, driver mutation status, initial stage, therapy line, liver metastasis, antibiotic use, PPI use, and *C. butyricum* therapy using CBM588, with each factor categorized as presented in Table 1. Propensity score adjustment helps preserve statistical power by reducing covariates into a single variable. To evaluate the adjusted effect of CBM588 or PPI, propensity scores were estimated. Propensity scores were calculated through binary logistic regression, predicting the probability of CBM588 or PPI use while accounting for background factors.

Subsequently, survival analyses were conducted using multivariate Cox proportional hazard models with inverse probability of treatment weighting (IPTW) using propensity scores, balancing relevant characteristics between the CBM588 and non-CBM588 groups or the PPI user and PPI non-user groups. To ensure statistical robustness, we implemented an alternative method employing propensity scores as covariates in multivariate Cox proportional hazards models. Statistical analyses were performed using R version 4.1.3 (The R Foundation for Statistical Computing, Vienna, Austria) [18,23], with significance set at *p* < 0.05. The Forester package (https://github.com/rdboyes/forester, latest accessed on 15 December 2023) was used to draw a forest plot. 

## 3. Results

### 3.1. Patient Characteristics

We retrospectively analyzed 106 patients with advanced or recurrent NSCLC consecutively treated with chemoimmunotherapy combinations. Six patients were excluded owing to insufficient clinical information. Among the 100 patients with NSCLC who received chemoimmunotherapy combinations, 72% were men, the median age was 67 years, and 45 patients (45%) received *C. butyricum* therapy using the live bacterial product CBM588. Fifty-five patients (55%) did not receive CBM588 (Table 1). Most patients (75%) received pembrolizumab-containing chemoimmunotherapy combinations as the first-line therapy. The median OS in our cohort was 20 months. The indications and characteristics of *C. butyricum* therapy using CBM588 are presented in Supplementary Table S1. Of the 100 patients, 45 (45%) received CBM588, among whom 38 (84%) received CBM588 concurrently with chemoimmunotherapy combinations (median duration, 12 months) and 7 (16%) received it before and during therapy (median duration, 15 months). CBM588 was administered to alleviate constipation (62%), nonspecific abdominal symptoms (20%), and idiopathic diarrhea (7%). CBM588 was prophylactically administered with antibiotics in three patients (7%). All patients who received CBM588 for constipation were also treated with laxatives such as magnesium oxide and/or sennoside A·B calcium. Moreover, 55 patients (55%) received a PPI within the 60-day window, and 46 (46%) received antibiotic therapy within 60 days prior to ICI therapy initiation. The characteristics of the PPI and antibiotic therapies are presented in Supplementary Tables S2 and S3, respectively. Of the 55 patients with NSCLC using a PPI, 24 were using esomeprazole (44%), 14 lansoprazole (25%), 14 vonoprazan fumarate (25%), 2 rabeprazole (4%), and 1 omeprazole (2%). β-lactam- and quinolone-based antibiotic therapies were the most commonly used antibiotics. In addition, 22/55 (38%), 24/45 (53%), 23/46 (50%), and 21/54 (41%) patients received CBM588 in the PPI user, PPI non-user (Table 1), antibiotic user, and antibiotic non-user groups, respectively. Among the 25 patients who received both PPIs and antibiotic therapy within the treatment window, 11 (44%) received CBM588.

### 3.2. Impact of CBM588 on the Efficacy of Chemoimmunotherapy Combinations

**Figure 1:**
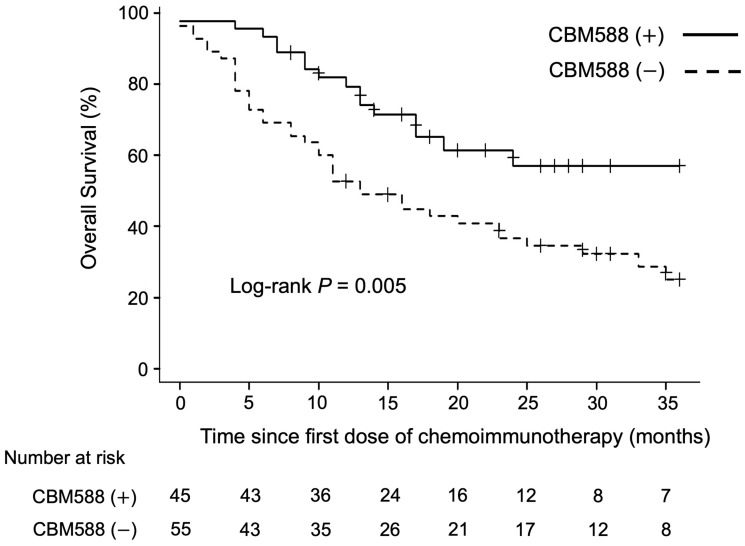
Association between *Clostridium butyricum* therapy using CBM588 and overall survival (OS) in patients with advanced non-small cell lung cancer treated with chemoimmunotherapy combinations. The figure illustrates the overall survival of patients, stratified by CBM588 administration.

We analyzed the association between *C. butyricum* therapy using CBM588 and OS in patients with NSCLC treated with chemoimmunotherapy combinations. The univariate analysis revealed that CBM588 was associated with longer PFS (HR 0.62, 95% CI 0.39–0.97, *p* = 0.036; log-rank test *p* = 0.032, median PFS [mPFS] 9 vs. 5 months) and OS (HR 0.44, 95% CI 0.25–0.79, *p* = 0.006; log-rank test *p* = 0.005, median OS [mOS]) not reached vs. 13 months, Figure 1).

The association of PFS and OS with patient characteristics in the chemoimmunotherapy combinations is shown in Table 2. According to the multivariable Cox proportional hazards regression model, the administration of CBM588 (HR 0.55, 95% CI 0.34–0.87, *p* = 0.011), sex (HR 0.54, 95% CI 0.33–0.88, *p* = 0.013), and liver metastasis (HR 3.33, 95% CI 1.69–6.58, *p* = 0.001) were independently associated with PFS (Table 2). The administration of CBM588 (HR 0.41, 95% CI 0.22–0.76, *p* = 0.004) and histology (HR 0.32, 95% CI 0.15–0.69, *p* = 0.003) were independently associated with OS (Table 3).

**Figure 2:**
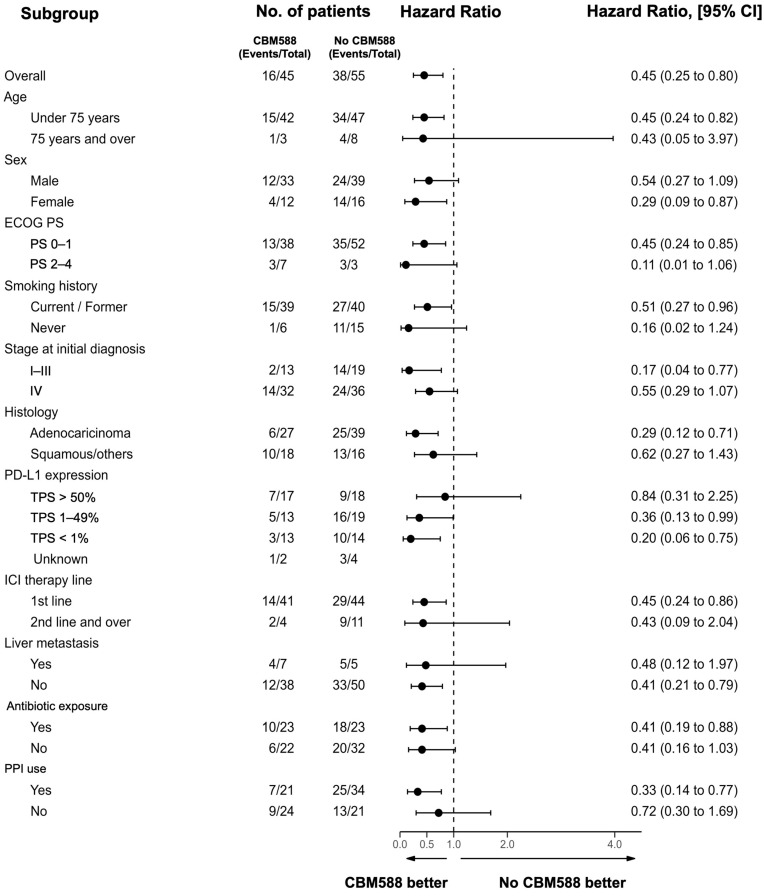
Subgroup analysis of overall survival among all patients. The dashed vertical line represents the hazard ratio. Factors considered include Eastern Cooperative Oncology Group performance status (ECOG PS), tumor proportion score (TPS), and proton pump inhibitors (PPIs).

Moreover, the multivariate Cox proportional hazards models with IPTW using propensity scores confirmed that CBM588 significantly prolonged PFS (IPTW-adjusted HR 0.55; *p* = 0.012; 95% CI, 0.35–0.88) and OS (IPTW-adjusted HR 0.36; *p* = 0.002; 95% CI, 0.19–0.69; Supplementary Table S4). To confirm the statistical robustness, we used the propensity score as a covariate in the Cox proportional hazards regression models and confirmed that CBM588 was independently associated with longer PFS (HR, 0.55; *p* = 0.027; 95% CI, 0.32–0.93) and OS (HR, 0.40; *p* = 0.005; 95% CI, 0.21–0.75). These results are consistent with those of our previous study in which we analyzed a cohort of patients who received single-agent ICI [23]. The results of the subgroup analyses conducted based on various clinicopathological factors associated with CBM588 (Figure 2) were consistent with those of the whole-cohort analyses, with the OS being superior in the CBM588 group in most analyses. In patients who were treated with chemoimmunotherapy combinations as a first-line treatment (*n* = 85), the administration of CBM588 led to a trend toward improved PFS (HR 0.64, 95% CI 0.39–1.05, *p* = 0.08; log-rank test *p* = 0.07, mPFS 9 vs. 4.5 months) and significantly prolonged OS (HR 0.45, 95% CI 0.24–0.85, *p* = 0.014; log-rank test *p* = 0.012, mOS not reached vs. 13 months) (Supplementary Figure S1).

### 3.3. Impact of CBM588 on Tumor PD-L1 Expression-Based OS

**Figure 3:**
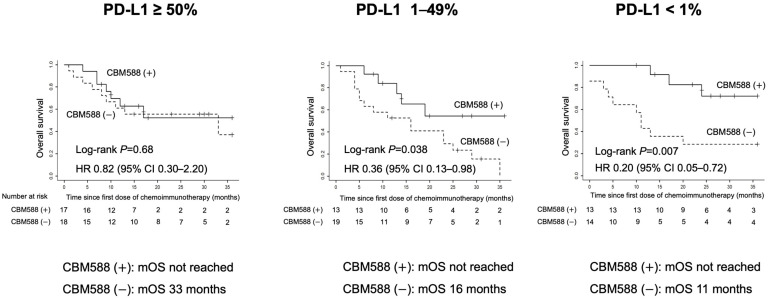
Kaplan–Meier estimates of a survival outcome in patients with advanced non-small cell lung cancer treated with chemoimmunotherapy combinations. The figure displays overall survival stratified by CBM588 administration. Kaplan–Meier curves depicting the impact of CBM588 based on tumor-programmed cell death ligand 1 (PD-L1) expression.

Tumor PD-L1 expression substantially affects the efficacy of chemoimmunotherapy combinations in NSCLC [2,3]; therefore, we investigated the effect of *C. butyricum* therapy using CBM588 on OS, based on PD-L1 expression in 94 patients for whom PD-L1 expression was determined. Kaplan–Meier curves displaying the impact of CBM588 based on tumor PD-L1 expression are presented in Figure 3.

Similar to the whole cohort, patients who received CBM588 exhibited significantly longer OS in the tumor PD-L1 < 1% (HR 0.20, 95% CI 0.05–0.72; log-rank test, *p* = 0.007, mOS not reached vs. 11 months, right panel) and PD-L1 1–49% (HR 0.36, 95% CI 0.13–0.98; log-rank test, *p* = 0.038, mOS not reached vs. 16 months, middle panel) cohorts. The positive impact of CBM588 in the PD-L1 <1% group was higher than that in the PD-L1 1–49% group. Conversely, in the PD-L1 ≥ 50% group, the OS did not significantly differ between the patients who received or did not receive CBM588 (HR 0.82, 95% CI 0.30–2.20; log-rank test, *p* = 0.68, mOS not achieved vs. 33 months, left panel).

### 3.4. CBM588 Enhances the Efficacy of Chemoimmunotherapy Combinations in Patients with NSCLC Receiving PPIs

We evaluated the effect of PPI use on patient survival in our cohort. The PPI use was not significantly associated with poor PFS (HR 1.46, 95% CI 0.93–2.29, *p* = 0.10, mPFS 4 versus 8 months) and OS (HR 1.30, 95% CI 0.76–2.24, *p* = 0.34, mOS 19 versus 23 months; Supplementary Figure S2).

**Figure 4:**
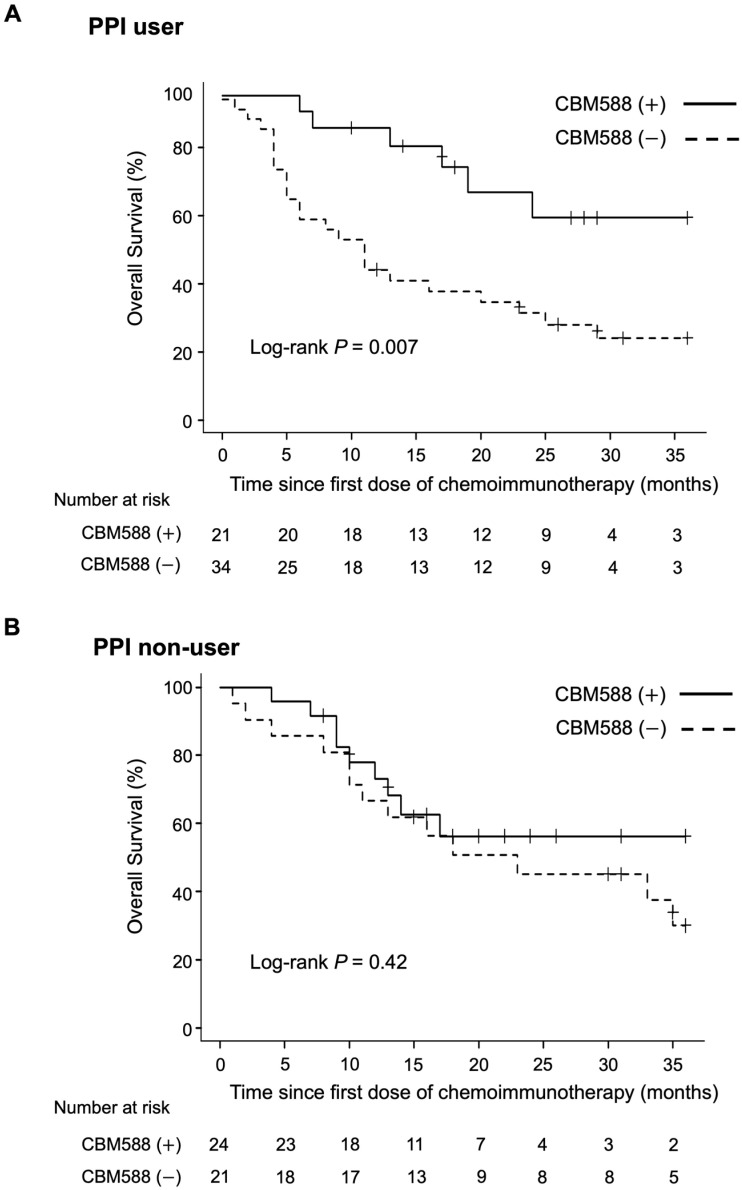
Kaplan–Meier estimates of a survival outcome in patients with non-small cell lung cancer (NSCLC) receiving proton pump inhibitors (PPIs) and those who did not receive PPIs within the 60-day window. (**A**) PPI user: overall survival (OS) in patients with NSCLC who received PPIs, stratified by CBM588 administration. (**B**) PPI non-user: OS of patients with NSCLC who did not receive PPIs, stratified by CBM588 administration.

Subsequently, we assessed the impact of CBM588 on survival in patients who received or did not receive PPIs within the 60-day window. In the PPI user cohort, patients administered CBM588 had significantly improved OS when compared with those not administered CBM588 (HR, 0.33; 95% CI 0.14–0.77; *p* = 0.010, log-rank test *p* = 0.007; mOS not reached vs. 11 months; Figure 4, upper panel); however the PFS in the patients administered CBM588 was not improved (HR, 0.64; 95% CI 0.35–1.17; *p* = 0.15, log-rank test *p* = 0.15; mPFS 7 vs. 4 months).

We conducted multivariate Cox proportional hazards models with IPTW using propensity scores. The propensity score analysis revealed significantly longer OS in patients who had received CBM588 than in those who had not (IPTW-adjusted HR, 0.26; 95% CI 0.10–0.64, *p* = 0.004; Supplementary Table S4); however, the PFS was not significantly improved (IPTW-adjusted HR, 0.59; 95% CI 0.32–1.07, *p* = 0.08). To confirm statistical robustness, we reanalyzed the data using the propensity score as a covariate in the Cox proportional hazards regression models, whichh confirmed that CBM588 was independently associated with longer PFS (HR 0.47, *p* = 0.039; 95% CI 0.23–0.96) and OS (HR 0.29, *p* = 0.006; 95% CI 0.12–0.70). Consistent with our earlier findings [18], CBM588 was not significantly associated with longer OS in the PPI non-user cohort (HR 0.70, 95% CI 0.30–1.66, *p* = 0.42, log-rank test *p* = 0.42, mOS not reached vs. 23 months; Figure 4, lower panel). These results suggest that CBM588 can potentially enhance the efficacy of chemoimmunotherapy combinations, especially in patients with cancer receiving PPIs.

### 3.5. Impact of CBM588 on Survival Outcomes in Patients with NSCLC Receiving Chemoimmunotherapy Combinations Alongside Antibiotics

Retrospective studies have confirmed that antibiotic exposure before ICI monotherapy is detrimental to clinical outcomes [5]. However, Cortellini et al. reported that prior antibiotic exposure did not impair the clinical outcomes of chemotherapy in patients with NSCLC [25]. Concordantly, antibiotic use within 60 days before initiating the chemoimmunotherapy combination was not significantly associated with worse PFS (HR 1.25, 95% CI 0.80–1.95, *p* = 0.33; log-rank test *p* = 0.33) and OS (HR 1.40, 95% CI 0.82–2.40, *p* = 0.22; log-rank test *p* = 0.21, Supplementary Figure S3) in our study.

**Figure 5:**
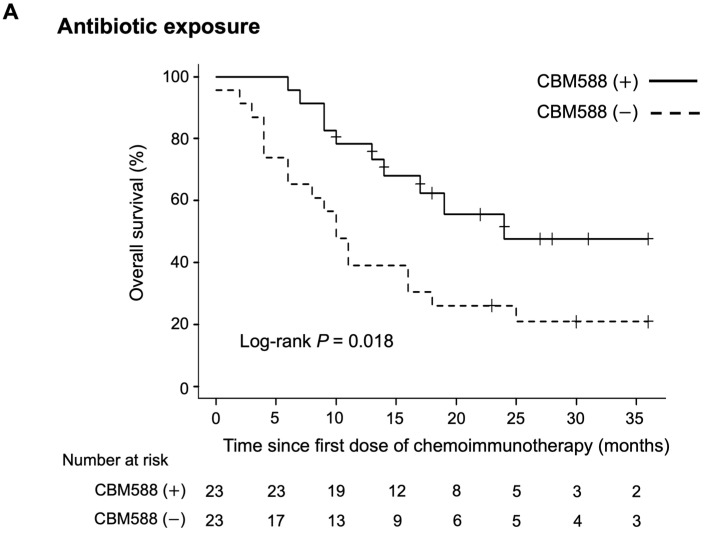

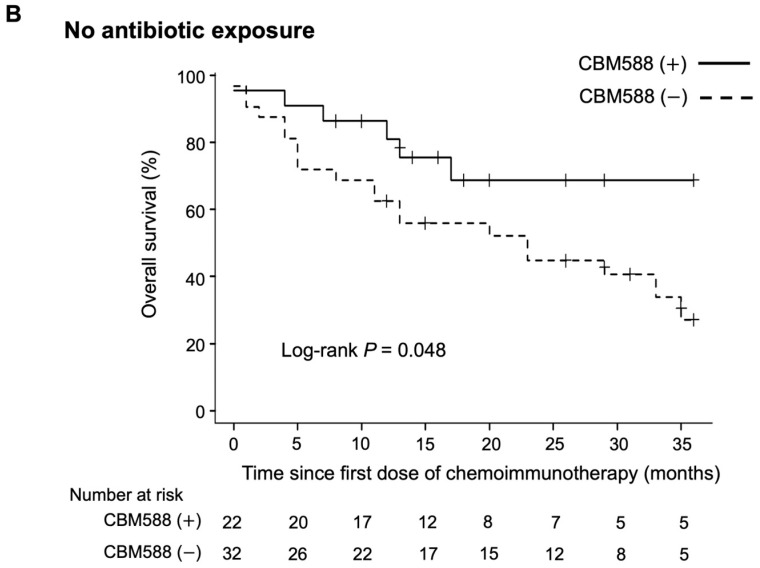
Kaplan–Meier estimates of survival outcome in patients with non-small cell lung cancer (NSCLC) who received antibiotics and those who did not receive antibiotics within the 60-day window. (**A**) Antibiotic exposure: overall survival (OS) in patients with NSCLC who received antibiotics, categorized by CBM588 administration. (**B**) No antibiotic exposure: OS of patients with NSCLC who did not receive antibiotics, stratified by CBM588 administration.

Subsequently, we evaluated the impact of CBM588 on the survival of patients who received antibiotics and those who did not. In patients who received antibiotic therapy within 60 days before the chemoimmunotherapy combination, patients administered CBM588 had longer PFS (HR 0.54, 95% CI 0.29–1.02, *p* = 0.06; log-rank test *p* = 0.018, mPFS 9 vs. 4 months) and OS (HR 0.40, 95% CI 0.18–0.87, *p* = 0.021; log-rank test *p* = 0.018, mOS 24 vs. 10 months; Figure 5, upper panel) than did those not administered CBM588. In patients who did not receive the antibiotic therapy, administration of CBM588 led to a trend toward improved PFS (HR 0.63, 95% CI 0.33–1.21, *p* = 0.16; log-rank test *p* = 0.16, mPFS 8.5 vs. 5 months) and OS (HR 0.41, 95% CI 0.16–1.02, *p* = 0.05; log-rank test *p* = 0.048, mOS not reached vs. 23 months; Figure 5, lower panel).

**Figure 6:**
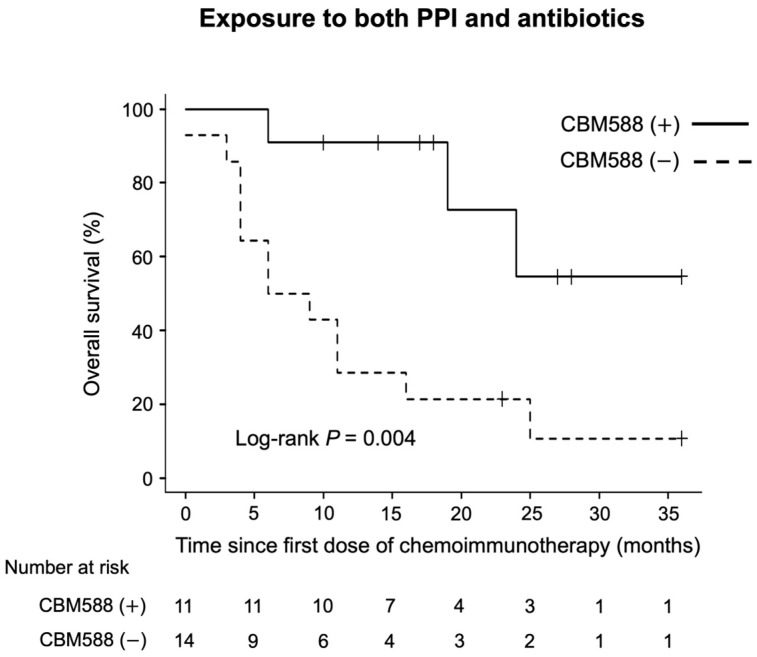
Kaplan–Meier estimate of overall survival (OS) in patients with non-small cell lung cancer (NSCLC) who received both PPIs and antibiotics within the 60-day window. The figure depicts the OS of patients with NSCLC treated with chemoimmunotherapy combinations, categorized by CBM588 administration.

Finally, we evaluated the impact of CBM588 on the survival of the 25 patients who received both PPIs and antibiotics within the therapeutic window. We observed a significant association between CBM588 and longer PFS (HR 0.38, 95% CI 0.15–0.92, *p* = 0.033; log-rank test *p* = 0.024, mPFS 9 vs. 3 months) and OS (HR 0.19, 95% CI 0.05–0.69, *p* = 0.011; log-rank test *p* = 0.005, mOS not reached vs. 7.5 months; Figure 6) in patients exposed to both antibiotics and PPIs.

## 4. Discussion

**Figure 7:**
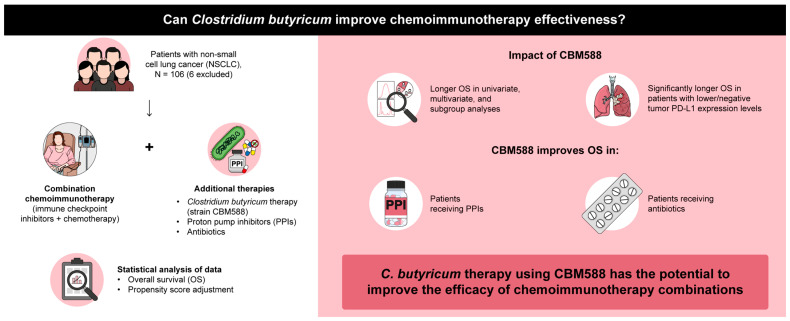
Overall schema of this study.

Mounting evidence suggests that the gut microbiota plays a pivotal role in influencing the response to cancer immunotherapy [5,6,8,10,28]. Recent clinical studies have demonstrated the potential of interventions, such as FMT and dietary modifications, in enhancing the effectiveness of ICIs [9,19,20]. However, the use of live biotherapeutic single bacterial strains for targeting gut microorganisms remains unestablished [5,21,29]. Additionally, treatments to restore the diminished therapeutic efficacy of ICIs in patients receiving gut dysbiosis-inducing medications, such as PPIs and antibiotics, remain unestablished [5,18,23]. Our current findings present compelling evidence for the positive impact of *C. butyricum* therapy using a single microbial strain, CBM588, on the efficacy of chemoimmunotherapy combinations in patients with NSCLC. CBM588 was significantly associated with prolonged OS in patients receiving chemoimmunotherapy combinations and significantly improved the OS in patients receiving both chemoimmunotherapy and dysbiosis-inducing drugs. These findings confirm that *C. butyricum* therapy using CBM588 may enhance the efficacy of chemoimmunotherapy combinations, thereby providing novel insights into the complex interplay among the gut microbiome, host immunity, and cancer immunotherapy (Figure 7).

The concomitant use of dysbiosis-inducing medications can disrupt the composition of the intestinal microbiota, which plays a pivotal role in regulating both innate and adaptive immune responses [18,29,30,31]. Previous research in preclinical mouse models and clinical investigations have emphasized the importance of the gut microbiota in modulating tumor responses to chemotherapeutic agents and ICIs [32,33,34,35,36]. Retrospective analyses of clinical studies and meta-analyses involving patients with advanced lung, kidney, and bladder cancers and melanoma have indicated a reduced clinical benefit of single-agent ICIs in patients exposed to antibiotics or PPIs around the time of initiation of ICIs [5,17]. We previously reported that the administration of CBM588, particularly in patients with NSCLC receiving PPIs and/or antibiotics, improved survival in a cohort primarily (>90%) receiving ICI monotherapy [18,23]. However, whether CBM588 can enhance the efficacy of chemoimmunotherapy combinations remains unconfirmed.

PPIs reportedly alter the gut microbiome’s composition by facilitating the translocation of oral commensals into the gut of patients with cancer, thereby modifying its immunomodulatory properties and compromising ICI efficacy [14,18,30,31]. We previously reported, by analyzing fecal samples from patients with lung cancer using 16S metagenomic sequencing, that patients receiving PPIs exhibited a higher abundance of harmful oral-related pathobionts and a lower abundance of beneficial gut bacteria crucial for immunotherapy. Additionally, patients receiving CBM588 had a reduced relative abundance of harmful oral-related bacteria in the gut. Consequently, we hypothesized that CBM588 enhances the outcomes of chemoimmunotherapy combinations by modulating the gut microbiota in patients using PPIs. In the present study, PPI use was not correlated with worse OS. This finding is inconsistent with that of a previous study based on a post hoc Cox proportional hazard analysis of a phase III trial, reporting that PPI use was associated with worse OS in patients treated with chemoimmunotherapy combinations, mirroring the impact of PPIs on survival in patients treated with ICI monotherapy [14]. Moreover, in the present study, CBM588 significantly improved the efficacy of chemoimmunotherapy combinations in patients receiving PPIs but not in those not receiving PPIs, which is consistent with our previous findings in a patient cohort predominantly (>90%) treated with ICI monotherapy [18]. These findings suggest that PPIs did not exert detrimental effects on the efficacy of chemoimmunotherapy combinations in our cohort and that the positive impact of CBM588 on patient survival was enhanced under conditions of PPI-induced gut dysbiosis, ultimately leading to improved survival in patients receiving PPI.

The PD-L1 TPS serves as a pivotal predictive biomarker for NSCLC patients most likely to benefit from ICI monotherapy and chemoimmunotherapy combinations [2]. In the present study, our subgroup analyses revealed that the influence of CBM588 on the efficacy of chemoimmunotherapy combinations varied depending on tumor PD-L1 expression in patients with NSCLC. Notably, CBM588 exhibited a positive impact on patients with NSCLC in the PD-L1 < 1% and PD-L1 1–49% groups but not in those with PD-L1 ≥ 50%. This result suggests that combining CBM588 with ICIs in NSCLC may yield maximum benefits in tumors with negative or low PD-L1 expression. γδ T cells, which possess properties of innate and adaptive immune cells, contribute to the response to ICIs in patients with HLA-class-I-negative cancers [37]. CBM588 might have stimulated innate-like γδ T cells and enhanced the efficacy of ICIs in the PD-L1 < 1% and PD-L1 1–49% groups, although the potential beneficial effects of CBM588 for clinical outcomes in patients with PD-L1 ≥ 50% may have been obscured by their high response to ICIs, coupled with the limited sample size.

Previous retrospective and prospective clinical studies on NSCLC and renal cell carcinoma have reported that CBM588 significantly enhances the efficacy of ICIs, although the underlying mechanisms remain elusive [18,22,23]. *C. butyricum* produces substantial amounts of butyric acid [38], and preclinical investigations have revealed that microbiota-derived butyrate enhances cellular metabolism and augments the memory potential of antigen-activated CD8^+^ T cells [39]. Additionally, butyrate, a microbial metabolite, can directly modulate antitumor CD8^+^ T cell responses and enhance chemotherapy effectiveness through ID2-dependent IL-12 signaling [40]. CBM588 also expands the population of resident *Bifidobacterium*, a potentially beneficial bacterium that promotes antitumor immunity and enhances the efficacy of ICIs [38,41]. Moreover, CBM588 directly induces the release of tumor necrosis factor-related apoptosis-inducing ligands from polymorphonuclear neutrophils, resulting in significant antitumor effects in vitro as well as in in vivo murine bladder cancer models [42]. These direct and indirect mechanisms by which the butyrate-producing human gut symbiont *C. butyricum* enhances host antitumor immunity may account for the positive impact of CBM588 on the efficacy of chemoimmunotherapy combinations.

We acknowledge some limitations in our study, including its retrospective design, small sample size, and cohort heterogeneity. Moreover, we did not investigate potential factors, such as diet, dietary fiber intake, lifestyle, and genetics, which could have influenced gut microbiota composition. In addition, the number of matching variables was high; thus, there is a potential risk of overmatching in our propensity score analyses. Despite these limitations, our findings represent the first evidence that manipulating the commensal microbiota using the live bacterial product CBM588 may enhance the activity of chemoimmunotherapy combinations. This study provides a compelling rationale for further validation through prospective studies.

## 5. Conclusions

We present novel evidence supporting the potential of CBM588-induced manipulation of commensal microbiota to enhance chemoimmunotherapy combination activity, which warrants further exploration of the synergy between CBM588 and immunotherapies.

## Figures and Tables

**Table 1:** Baseline characteristics of patients with advanced NSCLC receiving chemoimmunotherapy combinations.

	Total *n* = 100	CBM588 *n* = 45	No CBM588 *n* = 55	*p*-Value (CBM588 vs. No CBM588)
Median age (IQR)	67.0 (63.0–71.0)	67.0 (64.5–70.5)	66.0 (61.0–72.0)	0.57
Sex, *n* (%)				
Male	72 (72)	33 (73)	39 (71)	0.83
Female	28 (28)	12 (27)	16 (29)	
ECOG performance status, *n* (%)				
0–1	90 (90)	38 (84)	52 (95)	0.11
2–4	10 (10)	7 (16)	3 (5)	
Smoking history, *n* (%)				
Current/Former	79 (79)	39 (87)	40 (73)	0.14
Never	21 (21)	6 (13)	15 (27)	
Stage at initial diagnosis, *n* (%)				
I–III	32 (32)	13 (29)	19 (35)	0.67
IV	68 (68)	32 (71)	36 (65)	
Histology, *n* (%)				
Adenocarcinoma	66 (66)	27 (60)	39 (71)	0.29
Squamous/others	34 (34)	18 (12/6, 40%)	16 (12/4, 29%)	
EGFR/ALK mutation status, *n* (%)				
Wild-type	73 (73)	34 (76)	39 (71)	0.015
Mutant	11 (11)	1 (2)	10 (18)	
Unknown	16 (16)	10 (22)	6 (11)	
PD-L1 status, *n* (%)				
TPS ≥ 50%	35 (35)	17 (38)	18 (33)	0.85
TPS 1–49%	32 (32)	13 (29)	19 (35)	
TPS < 1%	27 (27)	13 (29)	14 (25)	
Unknown/Undeterminable	6 (6)	2 (4)	4 (7)	
Therapy line, *n* (%)				
1st line	85 (85)	41 (91)	44 (80)	0.16
≥2nd line	15 (15)	4 (9)	11 (20)	
Immune checkpoint inhibitor, *n* (%)				
Pembrolizumab	75 (75)	33 (73)	42 (76.4)	0.08
Atezolizumab	15 (15)	5 (11)	10 (18.2)	
Nivolumab + Ipilimumab	10 (10)	7 (16)	3 (5.4)	
Liver metastasis, *n* (%)				
Yes	12 (12)	7 (16)	5 (9)	0.37
No	88 (88)	38 (84)	50 (91)	
Antibiotic use, *n* (%)				
Yes	46 (46)	23 (51)	23 (42)	0.42
No	53 (54)	22 (49)	32 (58)	
PPI use, *n* (%)				
Yes	55 (55)	21 (47)	34 (62)	0.16
No	45 (45)	24 (53)	21 (38)	

Abbreviations: NSCLC, non-small cell lung cancer; IQR, interquartile range; ECOG, Eastern Cooperative Oncology Group; EGFR, epidermal growth factor receptor; *n*, number; PD-L1, programmed cell death ligand 1; TPS, tumor proportion score; PPI, proton pump inhibitor.

**Table 2:** Analyses of progression-free survival (PFS) in patients with NSCLC treated with chemoimmunotherapy combinations.

	Univariate Analysis		Multivariate Analysis	
Variables	HR (95% CI)	*p*-Value	HR (95% CI)	*p*-Value
CBM588				
No	Reference		Reference	
Yes	0.62 (0.39–0.97)	0.036	0.55 (0.34–0.87)	0.011
Age (years)	1.00 (0.97–1.03)	0.83		
Sex				
Female	Reference		Reference	
Male	0.59 (0.36–0.95)	0.030	0.54 (0.33–0.88)	0.013
ECOG performance status				
0–1	Reference			
2–4	1.83 (0.94–3.55)	0.08		
Smoking history				
Current/Former	0.75 (0.44–1.26)	0.28		
Never	Reference			
Stage at initial diagnosis				
I–III	Reference			
IV	1.31 (0.80–2.16)	0.28		
Histology				
Adenocarcinoma	0.70 (0.44–1.12)	0.14		
Squamous/others	Reference			
EGFR/ALK mutation status				
Wild-type	Reference			
Mutant	1.96 (0.99–3.88)	0.05		
Unknown	0.87 (0.45–1.66)	0.67		
PD-L1 status				
TPS < 1%	Reference			
TPS 1–49%	1.13 (0.65–1.98)	0.66		
TPS ≥ 50%	0.70 (0.39–1.27)	0.25		
Unknown/Undeterminable	1.20 (0.45–3.18)	0.71		
Therapy line				
1st line	Reference			
≥2nd line	1.34 (0.74–2.44)	0.34		
Liver metastasis				
Yes	2.54 (1.32–4.89)	0.005	3.33 (1.69–6.58)	0.001
No	Reference		Reference	
Antibiotic use				
Yes	1.25 (0.80–1.95)	0.33		
No	Reference			
PPI use				
Yes	1.46 (0.93–2.29)	0.10		
No	Reference			

Abbreviations: NSCLC, non-small cell lung cancer; IQR, interquartile range; ECOG, Eastern Cooperative Oncology Group; EGFR, epidermal growth factor receptor; PD-L1, programmed cell death ligand 1; TPS, tumor proportion score; PPI, proton pump inhibitor.

**Table 3:** Analyses of overall survival (OS) in patients with NSCLC treated with chemoimmunotherapy combinations.

	Univariate Analysis		Multivariate Analysis	
Variables	HR (95% CI)	*p*-Value	HR (95% CI)	*p*-Value
CBM588				
No	Reference		Reference	
Yes	0.44 (0.25–0.79)	0.006	0.41 (0.22–0.76)	0.004
Age (years)	1.00 (0.97–1.04)	0.94		
Sex				
Female	Reference			
Male	0.61 (0.35–1.08)	0.09		
ECOG performance status				
0–1	Reference			
2–4	1.64 (0.70–3.85)	0.26		
Smoking history				
Current/Former	0.92 (0.49–1.76)	0.81		
Never	Reference			
Stage at initial diagnosis				
I–III	Reference			
IV	1.13 (0.63–2.02)	0.69		
Histology				
Adenocarcinoma	0.53 (0.31–0.92)	0.024	0.32 (0.15–0.69)	0.003
Squamous/others	Reference		Reference	
EGFR/ALK mutation status				
Wild-type	Reference		Reference	
Mutant	2.42 (1.16–5.07)	0.019	2.13 (0.93–4.86)	0.07
Unknown	1.24 (0.61–2.51)	0.55	0.52 (0.22–1.26)	0.15
PD-L1 status				
TPS < 1%	Reference			
TPS 1–49%	1.59 (0.79–3.18)	0.19		
TPS ≥ 50%	1.10 (0.53–2.29)	0.80		
Unknown/Undeterminable	1.87 (0.61–5.76)	0.27		
Therapy line				
1st line	Reference			
≥2nd line	1.51 (0.78–2.93)	0.23		
Liver metastasis				
Yes	2.77 (1.32–5.82)	0.007	2.02 (0.90–4.53)	0.09
No	Reference			
Antibiotic use				
Yes	1.40 (0.82–2.40)	0.22		
No	Reference			
PPI use				
Yes	1.30 (0.76–2.24)	0.34		
No	Reference			

Abbreviations: NSCLC, non-small cell lung cancer; IQR, interquartile range; ECOG, Eastern Cooperative Oncology Group; EGFR, epidermal growth factor receptor; PD-L1, programmed cell death ligand 1; TPS, tumor proportion score; PPI, proton pump inhibitor.

## Data Availability

The data generated in this study are available upon request from the corresponding authors.

## Supplementary Materials

The following supporting information can be downloaded at: https://www.mdpi.com/article/10.3390/cancers16010047/s1, Figure S1: Association between *Clostridium butyricum* therapy using CBM588 and overall survival (OS) and progression-free survival (PFS) in patients who were treated with chemoimmunotherapy combinations as the first-line treatment (*n* = 85). The figures illustrate the OS (A) and PFS (B) of patients, stratified by CBM588 administration; Figure S2: Kaplan–Meier estimate of the overall survival (OS) for proton pump inhibitor (PPI) users vs. PPI non-users in patients with non-small cell lung cancer (NSCLC) receiving chemoimmunotherapy combinations. The figure illustrates the OS of patients with NSCLC receiving chemoimmunotherapy combinations, stratified by PPI usage within the 60-day window; Figure S3: Kaplan–Meier estimate of survival outcome for patients who received antibiotics vs. those who did not receive antibiotics within the 60-day window in patients with non-small cell lung cancer (NSCLC) receiving chemoimmunotherapy combinations. The figure displays the overall survival of patients with NSCLC receiving chemoimmunotherapy combinations, stratified by antibiotic exposure within the 60-day window; Table S1: Indications and characteristics of *Clostridium butyricum* therapy using CBM588; Table S2: Characteristics of PPIs within the 60-day window; Table S3: Characteristics of antibiotic therapy; Table S4: Cox proportional hazards regression models with inverse probability of treatment weighting using propensity score; CBM588 vs. no CBM588 in the total cohort and protein pump inhibitor user cohort.
