# Supplementary material for: Association of Clostridium butyricum Therapy Using the Live Bacterial Product CBM588 with the Survival of Patients with Lung Cancer Receiving Chemoimmunotherapy Combinations
Source: Cancers (Basel). 2023 Dec 21;16(1):47. doi: 10.3390/cancers16010047 (PMC10778075; doi:10.3390/cancers16010047)
Supplement: Supplementary file 1 [file cancers-16-00047-s001.zip › cancers-2776909-supplementary.pdf]

Supplementary Material

1 Supplementary Figures

A

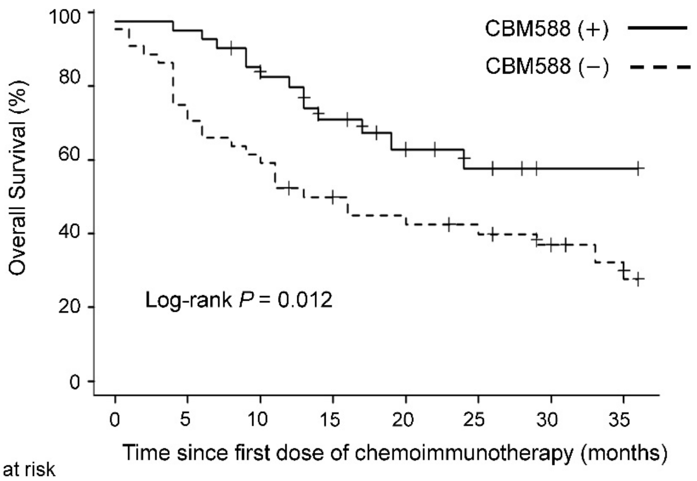

Number at risk

CBM588 (+)	41	39	33	21	14	10	7	7
CBM588 (−)	44	33	27	21	18	16	11	7

B

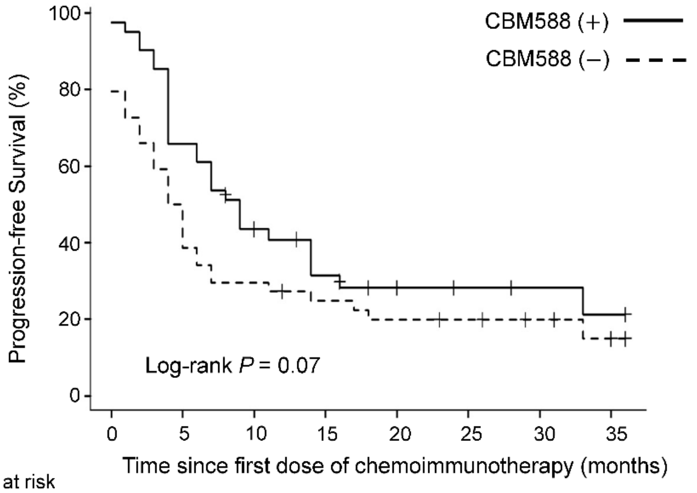

Number at risk

CBM588 (+)	41	27	17	10	7	5	4	3
CBM588 (−)	44	22	13	10	8	7	5	3

Supplementary Figure S1. Association between *Clostridium butyricum* therapy using CBM588 and overall survival (OS) and progression-free survival (PFS) in patients who were treated with chemoimmunotherapy combinations as the first-line treatment (n = 85). The figures illustrate the OS (A) and PFS (B) of patients, stratified by CBM588 administration.

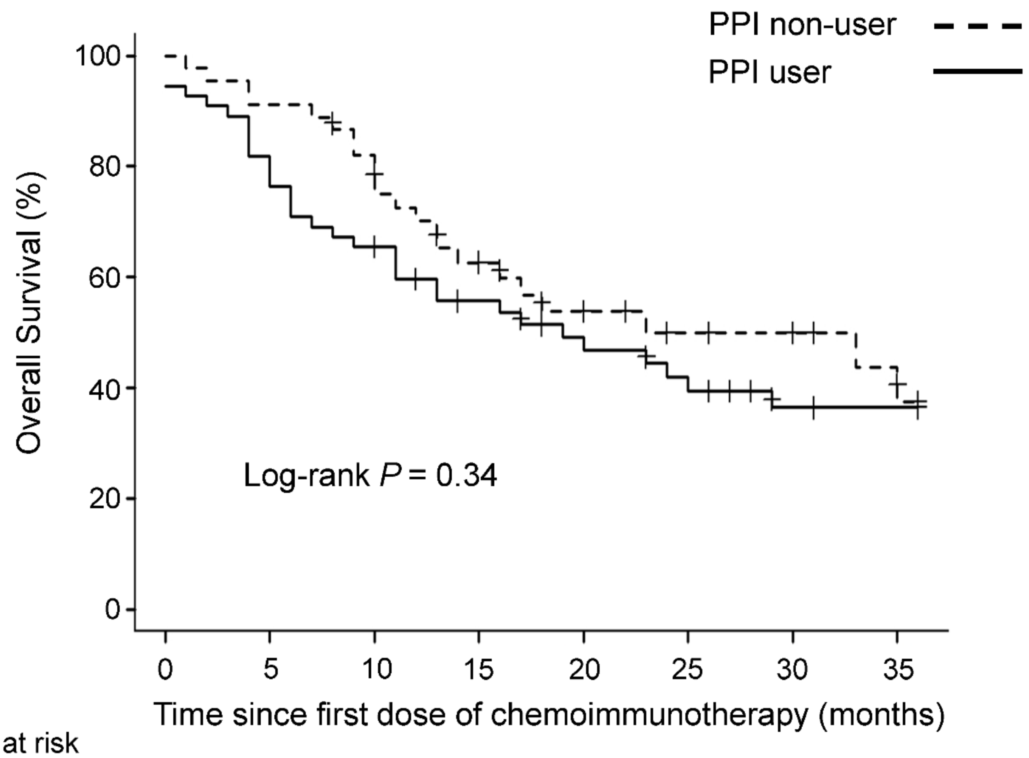

Number at risk

PPI non-user	45	41	35	24	16	12	11	7
PPI user	55	45	36	26	21	17	9	8

Supplementary Figure S2. Kaplan–Meier estimate of the overall survival (OS) for proton pump inhibitor (PPI) users vs. PPI non-users in patients with non-small cell lung cancer (NSCLC) receiving chemoimmunotherapy combinations. The figure illustrates the OS of patients with NSCLC receiving chemoimmunotherapy combinations, stratified by PPI usage within the 60-day window.

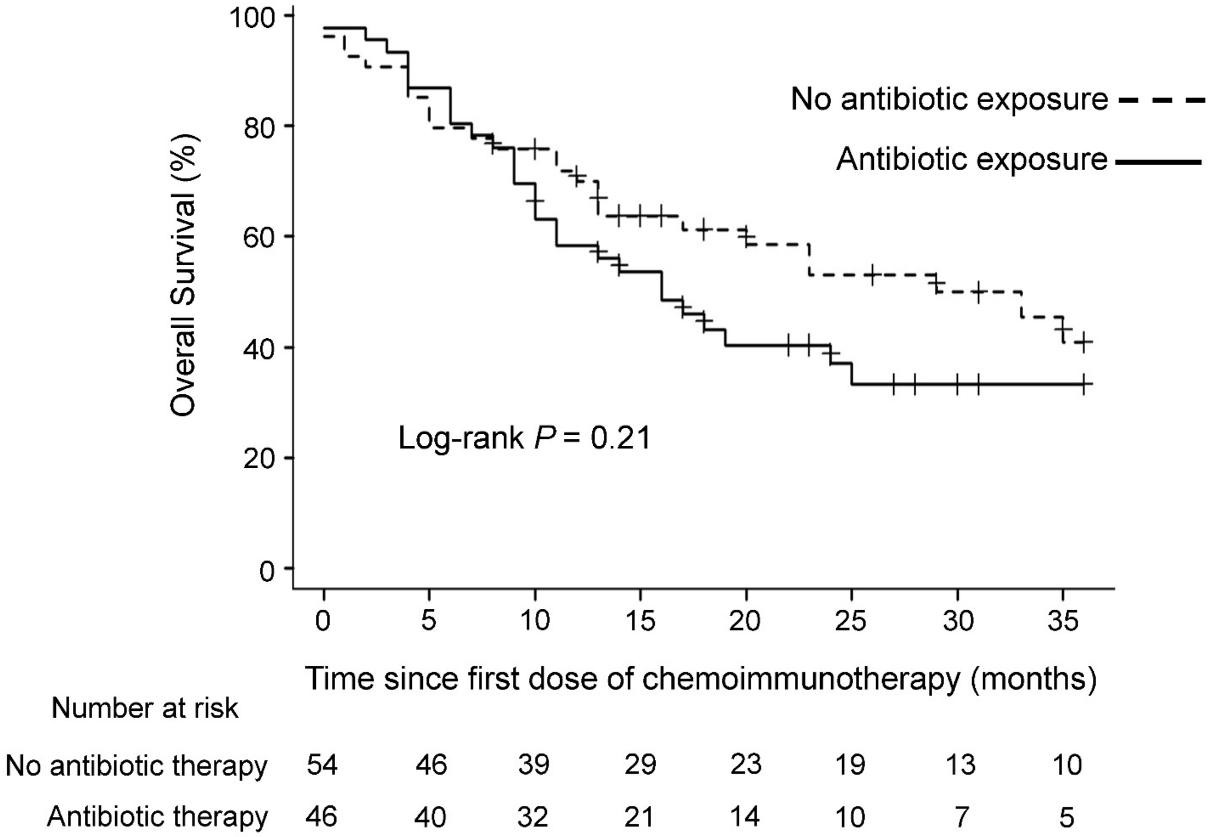

Supplementary Figure S3. Kaplan–Meier estimate of survival outcome for patients who received antibiotics vs. those who did not receive antibiotics within the 60-day window in patients with non-small cell lung cancer (NSCLC) receiving chemoimmunotherapy combinations. The figure displays the overall survival of patients with NSCLC receiving chemoimmunotherapy combinations, stratified by antibiotic exposure within the 60-day window.

2 Supplementary Tables

Supplementary Table S1. Indications and characteristics of *Clostridium butyricum* therapy using CBM588.

**Indication for CBM588[a]**	**Total** ***N*=45, (%)**	**Before ICI initiation** ***N*=0, (0%)**	**During ICI therapy** ***N*=38, (84%)**	**Before and during ICI therapy** ***N*=7, (16%)**
Idiopathic Diarrhea, *N* (%)	3 (7%)	0 (0%)	3 (7%)	0 (0%)
Constipation, *N* (%)	28 (62%)	0 (0%)	26 (58%)	2 (4.4%)
Non-specific abdominal symptoms, *N* (%)	9 (20%)	0 (0%)	6 (13%)	3 (7.2%)
Antibiotics-associated diarrhea, *N* (%)	1 (2%)	0 (0%)	1 (2%)	0 (0%)
Prophylactic administration with antibiotics, *N* (%)	3 (7%)	0 (0%)	1 (2%)	2 (4.4%)
Immune-related enterocolitis, *N* (%)	1 (2%)	0 (0%)	1 (2%)	0 (0%)
**Median dose (range)**	120 mg/day (40-120)	N/A	120 mg/day (60-120)	120 mg/day (40-120)
**Median duration of CBM588 (range)**	12 months (7 days-47 months)	N/A	12 months (7 days-47 months)	15 months (8 days-48 months)

Abbreviation: N/A, not applicable; ICI, immune checkpoint inhibitor. [a]Initial indications for CBM588 are shown.

Supplementary Table S2. Characteristics of PPIs within the 60-day window.

**PPIs**	**PPI user**<br>***N*=55, (%)**
Rabeprazole sodium	2 (4%)
Omeprazole	1 (2%)
Vonoprazan fumarate	14 (25%)
Esomeprazole magnesium hydrate	24 (44%)
Lansoprazole	14 (25%)

Supplementary Table S3. Characteristics of antibiotic therapy.

**Antibiotic class**	***N*=46**
β-lactams ± β-lactamase inhibitors	12
Carbapenems	1
Macrolides	1
Quinolones	17
Quinolones + β-lactams ± β-lactamase inhibitors	5
Tetracyclines + β-lactams	1
Quinolones + Sulfonamides	2
Tetracyclines	1
Sulfonamides	6

Supplementary Table S4. Cox proportional hazards regression models with inverse probability of treatment weighting using propensity score; CBM588 vs. no CBM588 in the total cohort and protein pump inhibitor user cohort.

CBM588 vs no CBM588 in total cohort: Hazard ratio for overall survival (OS)

OS (three years)	HR	95% CI	P-value
No CBM588 (Reference)			
CBM588	0.36	0.19-0.69	P = 0.002

Cox proportional hazards regression models with inverse probability of treatment weighting (IPTW) and robust standard errors were used to obtain hazard ratio (HR). The propensity score was calculated using the logistic regression model, in which age, sex, performance status, histology, smoking history, anti-bacterial agent, PPI medication, initial stage, ICI therapy line, driver mutation, metastasis of liver, and PD-L1 status were used as background factors. Each factor was categorized as shown in Table 1. Abbreviations: CI, Confidence interval; ICI, Immune checkpoint inhibitor; PD-L1, Programmed cell death ligand1

CBM588 vs no CBM588 in PPI user cohort: Hazard ratio for overall survival (OS)

OS (three years)	HR	95% CI	P-value
No CBM588 (Reference)			
CBM588	0.26	0.10-0.64	P = 0.004

Cox proportional hazards regression models with inverse probability of treatment weighting (IPTW) and robust standard errors were used to obtain hazard ratio (HR). The propensity score was calculated using the logistic regression model, in which age, sex, performance status, histology, smoking history, anti-bacterial agent, initial stage, ICI therapy line, driver mutation, metastasis of liver, and PD-L1 status were used as background factors. Each factor was categorized as shown in Ttable 1. Abbreviations: CI, Confidence interval; ICI, Immune checkpoint inhibitor; PD-L1, Programmed cell death ligand1
